# Peripheral Blood Eosinophilia in Patients with Diabetic Foot Infection Receiving Long-Term Antibiotic Therapy

**DOI:** 10.3390/jcm13072023

**Published:** 2024-03-30

**Authors:** Reut Kadosh Freund, Elimelech Rozenberg, Tali Shafat, Lisa Saidel-Odes

**Affiliations:** 1 The Faculty of Health Sciences, Ben-Gurion University of the Negev, P.O. Box 653, Beer-Sheva 84105, Israel; reutkad@clalit.org.il (R.K.F.); eliro@clalit.org.il (E.R.); talshaf@clalit.org.il (T.S.); 2 Clinical Immunology and Allergology, Soroka University Medical Center, P.O. Box 151, Beer-Sheva 84101, Israel; 3 Infectious Diseases Institute, Soroka University Medical Center, P.O. Box 151, Beer-Sheva 84101, Israel; 4 Infection Control and Hospital Epidemiology Unit, Soroka University Medical Center, P.O. Box 151, Beer-Sheva 84101, Israel

**Keywords:** drug-induced eosinophilia, diabetic foot infection, antibiotic therapy

## Abstract

**Background**: The eosinophil level in peripheral blood increases in response to various conditions, the most common being medication use. Since the outcome of increased levels of eosinophils can range from a benign finding to extensive damage to host organs and systemic consequences, this finding raises concern among clinicians. We aimed to assess the prevalence of prolonged antibiotic-therapy-induced eosinophilia and possible outcomes. **Methods**: We conducted a retrospective cohort study of diabetic patients admitted to the orthopedic department from December 2016 through December 2020 due to a moderate to severe diabetic foot infection and who received at least 14 days of antibiotic therapy. Patients were identified retrospectively through the orthopedic department registry, and their files were reviewed, extracting demographics, laboratory test results, antibiotic treatment, and outcomes. **Results**: The cohort included 347 patients; a total of 114 (32.8%) developed eosinophilia during the follow-up period. Patients who developed eosinophilia had a significantly longer duration of antibiotic treatment (*p* < 0.001) and a significantly longer hospitalization (*p* = 0.001). For multivariable analysis, the independent risk factors predicting drug-induced eosinophilia included older age, higher eosinophil count on admission (per quantile) and higher platelet count on admission (per quantile) (*p* = 0.012, *p* < 0.001, *p* = 0.009, respectively). There was no evidence of complications in patients who developed eosinophilia compared to patients who did not. No significant association with a specific type of antibiotic was found. **Conclusions**: We found a higher incidence of drug-induced eosinophilia than expected or previously described. The factors associated with eosinophilia included age and higher baseline eosinophil and platelet levels but not antibiotic type.

## 1. Introduction

Eosinophils are white blood cells (WBCs) of the granulocytic lineage involved in the host immune response to infection, tissue remodeling, tumor surveillance, and the maintenance of other immune cells [1]. In peripheral blood, an absolute eosinophil count of 0 to 500 cells/µL is typically considered normal. The degree of blood eosinophilia, in the absence of active treatment, can be categorized into mild (absolute eosinophil count 500–1500 cells/µL), moderate (absolute eosinophil count 1500–5000 cells/µL), or severe (absolute eosinophil count >5000 cells/µL) [2]. Hyper eosinophilia (≥1500 cells/µL) can affect organs such as the heart, the skin, the esophageal mucosa, the biliary tract, the central or peripheral nerves, and blood vessel walls, which might become pathological targets for eosinophil infiltration in a wide range of diseases. The airways also constitute a preferential target for eosinophil spreading during inflammation. After recruitment into inflamed tissues, eosinophils can cause damage by several mechanisms, yet those outcomes do not always occur [3,4]. As an increased eosinophil level may lead to a significant inflammatory response, various factors (other than infection) may lead to unwanted extensive eosinophilia. This in turn may have deleterious consequences with ensuing damage to healthy host tissues, sometimes with systemic consequences [4].

The most common cause of eosinophilia in developed countries is the use of drugs [5,6], with other causes, including helminthic infections, allergen exposure, and certain organ-specific diseases such as eosinophilic granulomatosis with polyangiitis, eosinophilic gastrointestinal disorders or nasal polyposis, or constitutively by malignant cells from solid tumors (usually adenocarcinomas), T-cell lymphomas, or Hodgkin’s lymphoma [7]. The main clinical entities included in the drug hypersensitivity reactions are isolated peripheral hyper-eosinophilia, maculopapular rash, and drug reaction with eosinophilia and systemic symptoms (DRESS). DRESS is characterized by a maculopapular rash accompanied by constitutional symptoms and multi-organ failure; in particular, the liver and the kidneys are frequently involved [4].

A previous study by Blumenthal et al. found that antibiotic-induced eosinophilia is common with parenteral antibiotics, especially during the use of vancomycin and penicillin. While most of these patients do not develop hypersensitivity reactions, eosinophilia is thought to increase the hazard rate of developing a rash and renal injury [8]. The latency period between drug exposure and the development of eosinophilia is highly variable and can range from hours to weeks, thereby complicating the identification of the culprit insult [9].

Our research focused on diabetic patients hospitalized due to a diabetic foot infection. This complication occurs in 6.4% of diabetic patients and is associated with major morbidity and increased mortality [10,11]. Patients with local or systemic infections require management that includes hospitalization for prolonged parenteral antibiotic treatment, often accompanied by a surgical intervention, followed by a step-down to oral therapy once the patient is stable and the infection has not progressed [12,13].

We aimed to assess the prevalence of drug-induced eosinophilia in a cohort of diabetic patients who were hospitalized for a diabetic foot infection, receiving a range of long-term antibiotic treatments, and at risk for possible complications such as a rash, renal injury, liver injury, and hypersensitivity reactions.

## 2. Methods

### 2.1. Study Population

We scanned electronic medical records to identify all adult patients hospitalized in the orthopedic department at Soroka University Medical Center for a diabetic foot infection, from December 2016 to December 2020. Eligible patients were those who had a diabetic foot ulcer grade ≥ 3, according to the International Working Group of the Diabetic Foot/Infectious Diseases Society of America classification for foot infections in persons with diabetes [13] requiring treatment with an antibiotic (oral/intravenous) for at least two consecutive weeks. Definitive antibiotic treatment was prescribed according to microbiological data, based on wound specimen cultures, which included bone biopsies in 74% of patients and deep tissue biopsies or pus in 25% of patients [10]. Exclusion criteria included patients with eosinophilia (as defined below) on their admission baseline complete blood count (CBC) drawn on the day of hospitalization, patients treated with immunomodulatory drugs (e.g., corticosteroids, colchicine), and those with a chronic diagnosis of asthma or atopic allergy. Patients were assessed for laboratory test results at two-week intervals during a follow-up period of up to six weeks, starting from admission. Patients included in the analysis had at least two differential CBC measurements taken on admission and two weeks later. Patients were followed by the infectious diseases service during hospitalization and post-discharge in the setting of an outpatient infectious diseases clinic up to six weeks following discharge, parallel to their follow-up in the orthopedic clinic.

Data collected from electronic patients’ files included demographics, intravenous and oral antibiotics administered, treatment duration, laboratory tests including the absolute eosinophils count, renal function, liver enzymes, inflammatory markers; and possible complications such as rash, recurrent hospitalizations, and mortality. All decisions related to antibiotic change in relation to culture results, including medication type and duration, were determined by the primary consultant infectious diseases physician of the department.

### 2.2. Eosinophilia Definition

We defined eosinophilia as any absolute eosinophil count greater than or equal to 500 cells/µL that appeared two weeks or more after treatment initiation. Absolute eosinophil count above 1500 cells/µL was defined as hyper-eosinophilia.

### 2.3. Antibiotics-Induced Eosinophilia Outcomes

Electronic patient files were further reviewed for appearance of a new rash during admission and follow-up, including dermatology consultation, family physician visits, and outpatient clinic visits. Renal injury was represented by a change in estimated Glomerular Filtration Rate (eGFR), using the MDRD formula. Liver injury was measured by increased levels of Alanine Transaminase (ALT) and Aspartate Aminotransferase (AST) above 34 and 31 U/L, respectively. Elevated laboratory inflammatory markers were defined by a C-reactive protein level above 0.5 mg/dL and a platelet level above 400 × 10^3^/uL. Recurrent hospitalization for a DFI was defined as a second admission within six months of the previous discharge.

### 2.4. Statistical Analysis

Categorical data were expressed as absolute numbers and percentages, continuous variables as mean (SD), and ordinal with non-parametric distribution as median and interquartile range. Differences between characteristics of patients that developed drug-induced eosinophilia and those free of drug-induced eosinophilia were assessed by Student’s *t*-test for continuous variables, Mann–Whitney test for variables with non-parametric distribution, and chi-square test (x^2^) for categorical variables. Risk factors for drug-induced eosinophilia among the study population were assessed using logistic regression and were described as 95% CI. The association between drug-induced eosinophilia and eGFR was assessed using linear regression analysis and described as *β* (slope) and 95% CI. Two-sided *p*-values < 0.05 were considered statistically significant. All statistical analyses were conducted using SPSS 25.0 statistical software (IBM Corp Armonk, New York, NY, USA).

## 3. Results

**Figure 1:**
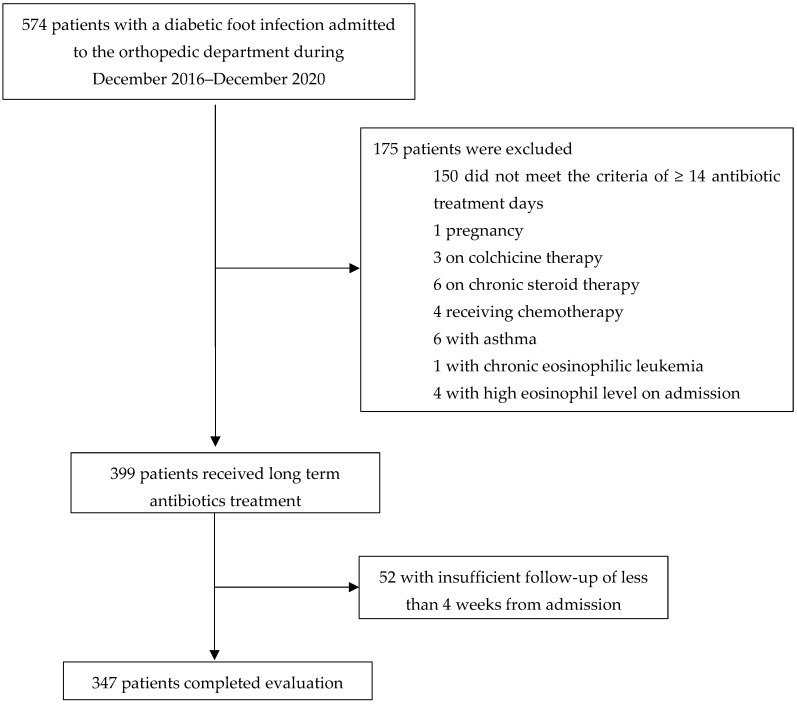
Research population.

From December 2016 through December 2020, 574 patients were admitted to the orthopedic department for treatment of a diabetic foot infection, of whom 347 met the inclusion criteria (Figure 1). Patients’ glycemic control on admission was poor, with an HbA1C of 8.1, 7.0–10.2 (median, IQR).

Patients’ mean age was 61.9 years ± 12.1, and 74.4% were male (Table 1), 75% were Jewish, and 25% were of Bedouin Arab ethnicity. Eosinophilia developed in 114 of 347 patients (32.8%) over 2 to 6 weeks after treatment initiation. The sociodemographic characteristics of those who developed eosinophilia and those who did not were similar, except for their age. Patients who developed eosinophilia were older (63.9 ± 11.7 vs. 60.9 ± 12.1, *p* = 0.031), and their eosinophil median level on admission was higher than in those that did not later develop eosinophilia (166.5 ± 116.8 vs. 111.3 ± 91.5, *p* < 0.001), though it was in a normal range (<500) for both groups (Table 1).

The median duration of antibiotic therapy was 39 days (IQR, 26–47 days) in the whole cohort, with a longer median duration of antibiotic treatment among patients who developed eosinophilia (42 vs. 35 days, *p* < 0.001) (Table 2).

No specific antibiotic type was found to be associated with a higher risk of developing eosinophilia while administered for ≥14 consecutive days (Table 2). In contrast, trimethoprim-sulfamethoxazole was associated with reduced risk in the multivariable model analysis (0.11, 95% CI, 0.01–0.86) (Table 3 and Table S1).

On multivariable analysis, a higher eosinophil count on admission and a higher platelet count on admission (per quantile) were associated with an increased risk of developing drug-induced eosinophilia. Older age was mildly associated with an increased risk of developing drug-induced eosinophilia. However, hemodialysis therapy seemed to reduce the risk of developing eosinophilia (OR 0.39, 95% CI 0.17–0.92).

Among patients who developed eosinophilia, there was no significant impact on renal or liver function, and none developed a rash. Nevertheless, we found a higher platelet count during follow-up in this group (404.6 vs. 372.9, *p* = 0.037) and an extended hospitalization length (18 vs. 15 days, *p* = 0.001). We did not find a higher incidence of recurrent hospitalizations (Table 4).

While analyzing only patients who displayed worsening renal function at weeks 4–6 according to eGFR values, we found an association with vancomycin treatment (β −0.6, 95% CI −39.37–(−9.08), *p* = 0.002) but no impact of drug-induced eosinophilia (Table S2).

Among patients who developed drug-induced eosinophilia, there was a small group of 13 patients (3.7% of the study population) with hyper-eosinophilia (absolute eosinophil count > 1500 cells/µL). The characteristics of this group are consistent with older age (mean 67.6 years) and a higher level of eosinophils on admission, though within the normal range (mean 206.9 cells/µL), and nearly half of them (46.2%) had an additional hospitalization during the three months prior to the current admission (Table S3).

## 4. Discussion

We assessed a unique population of patients with diabetes mellitus hospitalized for a moderate to severe diabetic foot infection and found that eosinophilia occurred in a third of patients, although the eosinophilia was of no clinical consequence. We found the risk for developing eosinophilia was higher with age, a higher eosinophil count on admission, and a higher platelet count on admission. We did not find a higher risk for eosinophilia development in relation to exposure to a specific antibiotic type.

Our finding that one third of patients on prolonged antibiotic treatment (intravenous with/without oral) developed drug-induced eosinophilia is higher than expected and previously described. In a previous study by Blumenthal et al., only 25% of patients receiving intravenous antibiotic therapy as outpatients developed eosinophilia [8]. The comorbidities of the patients in Blumenthal’s study were not mentioned. In our patient population, all patients were diabetic. An additional difference was the most commonly treated infections and therefore the most commonly used antibiotics. All our patients were treated for a diabetic foot infection and the most common antibiotics used were penicillins (42%), cephalosporins (40%), and quinolones (26%). In Blumenthal’s study, the most common infections treated were orthopedic (not limited to diabetic foot infections) and bacteremia, and the most common antibiotics used were cephalosporins (46%), vancomycin (40%), and penicillins (27%). These differences might explain the higher-than-expected eosinophilia in our unique patient population.

Our results did not indicate an association between a certain antibiotic type with an increased hazard of developing eosinophilia. Penicillin and cephalosporins, both beta-lactam-based antibiotics and highly efficient antibiotics for the management of diabetic foot infections, were not found to be associated with an increased rate of eosinophilia. Interestingly, in our study, the 39 (11%) patients who received vancomycin did not develop eosinophilia, even though this antibiotic is known for its ability to cause a variety of hypersensitivity reactions such as maculopapular rash, interstitial nephritis, and even DRESS syndrome, all of which can include peripheral blood eosinophilia [5,14,15,16]. We did find that vancomycin increased the hazard rate of renal injury, a known adverse event [17,18]. The only antibiotic that decreased the risk of developing eosinophilia was trimethoprim-sulfamethoxazole. Like vancomycin, it is typically connected to adverse reactions such as DRESS syndrome, rash, Stevens–Johnson syndrome, toxic epidermal necrolysis, and interstitial nephritis, all of which can also present with eosinophilia. On the other hand, trimethoprim-sulfamethoxazole has also been described as causing pancytopenia [19,20].

While we could not show an association with a particular antibiotic, we found that the risk of developing eosinophilia was higher in patients of older age, especially when admitted with eosinophil levels at the upper range of the normal limits. Among patients who developed eosinophilia, we noticed a higher platelet level on admission and in the follow-up period, which can be another indication of an increased inflammatory state that may explain the significantly extended period of treatment and hospitalization in our patient population. Nevertheless, there was no statistical significance of elevated C-reactive protein levels between patients who developed eosinophilia compared to those who did not. This lack of association may be explained by our definition of an elevated C-reactive protein as 0.5 mg/dL and higher, while generally in major infections we will expect much higher levels.

Unique to Southern Israel is its diverse population; Bedouin Arabs, a formerly nomadic people, have rapidly urbanized in the last half century, accounting for about 25% of Southern Israel’s population. The age-adjusted prevalence of diabetes is 12.3% in the Bedouin Arab population compared to 8.2% in the Jewish population in Southern Israel [21]. The proportion of patients of Bedouin Arab ethnicity hospitalized due to a DFI in our orthopedic department increased from 21.8% to 27.8% of all diabetic foot infection admissions in the past decade. This is in accordance with the increasing prevalence of diabetes in the Bedouin Arab population in Southern Israel. Of note, we found no difference in eosinophilia development between the two populations.

Most importantly, our results show that drug-induced eosinophilia had no clinical consequence such as renal or liver injury, nor an increase in the incidence rate of rash development. These results suggest a reassuring message for clinicians. Yet, considering the potentially disturbing consequences of eosinophilia, every case should be closely examined.

Our study has some limitations. Firstly, our research is a retrospective population-based cohort study; therefore, we can only use information that was entered into the data system. Since the rash variable depends on the attention and reporting of physicians, we cannot exclude under-reporting. Secondly, considering the small sample of patients who received certain antibiotic types, significant differences might not have been apparent, and further research in a larger cohort is needed. Thirdly, we cannot be absolutely certain that the eosinophilia observed may not be solely due to drug-induced eosinophilia. Fourth, we did not collect data regarding a previous history of cardiovascular disease or peripheral vascular disease, which might influence inflammatory markers as we focused on diabetes mellitus and infection wound severity, which by themselves greatly influence the inflammatory markers and probably have a greater contribution to a possible association with eosinophilia response during infection.

## 5. Conclusions

In conclusion, while we found a higher incidence of drug-induced eosinophilia than previously described, this did not appear to have a significant clinical effect. This would support the continuation of antibiotic treatment even for patients with a diabetic foot infection who develop mild eosinophilia. However, older age and a higher eosinophil count on admission should alert physicians to the increased risk in this population.

## Figures and Tables

**Table 1:** Sociodemographic and baseline clinical and laboratory characteristics of the patient population; those that developed eosinophilia vs. those that did not develop eosinophilia.

Variable	Total (*n* = 347)	Eosinophilia (*n* = 114)	No Eosinophilia (*n* = 233)	*p*-Value
Age, years (mean ± SD)	61.9 ± 12.1	63.9 ± 11.7	60.9 ± 12.1	0.031
Male Gender, N (%)	258 (74.4)	86 (75.4)	172 (73.8)	0.746
Ethnicity				0.367
Jewish, N (%)	260 (74.9)	82 (71.9)	178 (76.4)	
Bedouin Arab, N (%)	87 (25.1)	32 (28.1)	55 (23.6)	
Body mass index (mean ± SD)	27.8 ± 5.5	27.9 ± 5.8	27.8 ± 5.4	0.837
Previous hospitalization in the past 3 months, N (%)	80 (23.1)	33 (28.9)	47 (20.2)	0.068
Hypertension, N (%)	262 (75.5)	89 (78.1)	173 (74.2)	0.437
Hemodialysis treatment, N (%)	41 (11.8)	8 (7.0)	33 (14.2)	0.053
Eosinophil count on admission (mean ± SD)	129.4 ± 103.7	166.5 ± 116.8	111.3 ± 91.5	<0.001
Platelet count on admission (mean ± SD)	322.2 ± 117.9	348.4 ± 133.6	309.4 ± 107.5	0.004
Body mass index (mean ± SD)	27.8 ± 5.5	27.9 ± 5.8	27.8 ± 5.4	0.837

**Table 2:** Long-term antibiotic therapy characteristics of patients with and without eosinophilia.

Variable	Total (*n* = 347)	Eosinophilia (*n* = 114)	No Eosinophilia (*n* = 233)	*p*-Value
Treatment duration (days), median, IQR	39, 26–47	42, 32–50	35, 23–44	<0.001
Vancomycin, N (%)	39 (11.2)	11 (9.6)	28 (12.0)	0.512
Narrow spectrum penicillin ^1^, N (%)	54 (15.6)	17 (14.9)	37 (15.9)	0.815
Broad spectrum penicillin ^2^, N (%)	90 (25.9)	36 (31.6)	54 (23.2)	0.093
Cephalosporins 1st–2nd generation, N (%)	92 (26.5)	27 (23.7)	65 (27.9)	0.404
Cephalosporins 3rd generation, N (%)	46 (13.3)	14 (12.3)	32 (13.7)	0.708
Quinolones, N (%)	89 (25.6)	29 (25.4)	60 (25.8)	0.950
Monobactam, N (%)	4 (1.2)	3 (2.6)	1 (0.4)	0.071
Metronidazole N (%)	19 (5.5)	9 (7.9)	10 (4.3)	0.166
Trimethoprim-sulfamethoxazole N (%)	13 (3.7)	1 (0.9)	12 (5.2)	0.049
Clindamycin N (%)	10 (2.9)	2 (1.8)	8 (3.4)	0.380

^1^ Narrow-spectrum penicillin included penicillin G, ampicillin, and amoxicillin. ^2^ Broad-spectrum penicillin included piperacillin/tazobactam, meropenem, and ertapenem.

**Table 3:** Multivariable analysis (logistic regression) for drug-induced eosinophilia.

Variable	OR	95% CI	*p*-Value
Age	1.03	1.01–1.05	0.012
Hemodialysis therapy	0.39	0.17–0.92	0.032
Previous hospitalization in the past 3 months	1.74	0.99–3.041	0.054
Eosinophil count on admission (quintiles)	1.44	1.22–1.72	<0.001
Platelet count on admission (quintiles)	1.26	1.06–1.50	0.009
Trimethoprim-sulfamethoxazole therapy	0.11	0.01–0.86	0.036

**Table 4:** Laboratory test results and outcomes of patients with and without eosinophilia within 6 weeks of admission.

Variable	Total (*n* = 347)	Eosinophilia (*n* = 114)	No Eosinophilia (*n* = 233)	*p*-Value
Minimal eGFR (mean ± SD) during follow-up	67.0 ± 42.1	66.4 ± 41.0	67.3 ± 42.7	0.849
Maximal C-reactive protein (median, IQR) during follow-up	5.5, 1.8–12.2	5.7, 2.1–12.5	5.2, 1.7–11.9	0.530
Maximal platelet count during follow-up (mean ± SD)	383.3 ± 133.3	404.6 ± 131.7	372.9 ± 133.1	0.037
New rash N (%)	1 (0.3)	0 (0)	1 (0.4)	0.484
Length of hospitalization stay (days) median, IQR	16, 10–24	18, 11–30	15, 9–21	0.001
Recurrent hospitalization within 6 months post-discharge N (%)	147 (42.4)	44 (38.6)	103 (44.2)	0.321

## Data Availability

The datasets generated and/or analyzed during the current study are not publicly available but are available from the corresponding author upon reasonable request.

## Supplementary Materials

The following supporting information can be downloaded at: https://www.mdpi.com/article/10.3390/jcm13072023/s1, Table S1. Antibiotic type given to DFI patients with and without eosinophilia while receiving long-term therapy (*n* = 347). Table S2. Multivariable analysis for renal function at weeks 4–6 according to eGFR (MDRD) (linear regression). Table S3. Characteristics of patients with hyper-eosinophilic syndrome (absolute eosinophil count > 1500 cells/µL) (*n* = 13).
