# Supplementary material for: Peripheral Blood Eosinophilia in Patients with Diabetic Foot Infection Receiving Long-Term Antibiotic Therapy
Source: J Clin Med. 2024 Mar 30;13(7):2023. doi: 10.3390/jcm13072023 (PMC11012316; doi:10.3390/jcm13072023)
Supplement: Supplementary file 1 [file jcm-13-02023-s001.zip › jcm-2912436-supplementary.pdf]

Supplementary Materials

Table S1. Antibiotic type given to DFI patients with and without eosinophilia while receiving long-term therapy (n=347).

Variable	Total (n=347)	Eosinophilia (n=114)	No eosinophilia (n=233)	P- value
Vancomycin, N (%)	39 (11.2)	11 (9.6)	28 (12.0)	0.512
Penicillin				
Any penicillin N (%)	137 (39.5)	49 (43.0)	88 (37.8)	0.351
Amoxicillin N (%)	10 (2.9)	3 (2.6)	7 (3.0)	0.845
Ampicillin N (%)	22 (6.3)	6 (5.3)	16 (6.9)	0.565
Amoxicillin/clavulanate PO N (%)	18 (5.2)	7 (6.1)	11 (4.7)	0.576
Amoxicillin/clavulanate IV N (%)	4 (1.2)	1 (0.9)	3 (1.3)	0.737
Piperacillin/tazobactam, N (%)	42 (12.1)	18 (15.8)	24 (10.3)	0.141
Cephalosporins				
Any cephalosporin N (%)	137 (39.5)	41 (36.0)	96 (41.2)	0.349
1st generation IV N (%)	45 (13.0)	15 (13.2)	30 (12.9)	0.941
1st generation PO N (%)	41 (11.8)	9 (7.9)	32 (13.7)	0.113
2nd generation IV N (%)	7 (2.0)	4 (3.5)	3 (1.3)	0.167
Ceftriaxone N (%)	41 (11.8)	11 (9.6)	30 (12.9)	0.382
Ceftazidime N (%)	5 (1.4)	3 (2.6)	2 (0.6)	0.336
Carbapenems				
Meropenem N (%)	39 (11.2)	16 (14.0)	23 (9.9)	0.249
Ertapenem N (%)	9 (2.6)	2 (1.8)	7 (3.0)	0.491
Quinolones				
Ciprofloxacin IV N (%)	10 (2.9)	5 (4.4)	5 (2.1)	0.241
Ciprofloxacin PO N (%)	77 (22.2)	24 (21.1)	53 (22.7)	0.721
Levofloxacin PO N (%)	3 (0.9)	1 (0.9)	2 (0.9)	0.986
Monobactam, N (%)	4 (1.2)	3 (2.6)	1 (0.4)	0.071
Others				
Metronidazole N (%)	19 (5.5)	9 (7.9)	10 (4.3)	0.166
Linezolid N (%)	3 (0.9)	3 (2.6)	0 (0)	0.013
Trimethoprim-sulphamethoxazole N (%)	13 (3.7)	1 (0.9)	12 (5.2)	0.049
Clindamycin N (%)	10 (2.9)	2 (1.8)	8 (3.4)	0.380

Table S2. Multivariable analysis for renal function at weeks 4-6 according to eGFR (MDRD) (linear regression) .

Variable	β	95% CI	P- value
Age	-0.31	-1.61- (-0.82)	<0.001
Male Gender	0.05	-5.45-16.25	0.328
Drug induced eosinophilia	0.04	-6.66-13.66	0.499
Vancomycin treatment	-0.16	-39.37-(-9.08)	0.002

Table S3. Characteristics of patients with hyper-eosinophilic syndrome (absolute eosinophil count > 1500 cells/μL) (n=13):.

	Variable	
	**Age (mean ±SD)**	67.6±11.8
	Male Gender, N (%)	12 (92.3)
Ethnicity	Jewish, N (%)	10 (76.9)

Bedouin Arab, N (%)	3 (23.1)
Body mass index (mean ±SD)	26.7±5.5
Previous hospitalization in the past 3 months N (%)	6 (46.2)
Hypertension, N (%)	11 (84.6)
Hemodialysis treatment, N (%)	2 (15.4)
Antibiotic type	
Vancomycin, N (%)	1 (7.7)
Narrow spectrum penicillin, N (%)	2 (15.4)
Wide spectrum penicillin, N (%)	3 (23.1)
Cephalosporins 1st and 2nd generation, N (%)	1 (7.7)
Cephalosporins 3rd generation, N (%)	3 (23.1)
Quinolones, N (%)	2 (15.4)
Monobactam, N (%)	2 (15.4)
Metronidazole N (%)	1 (7.7)
Labs	
Eosinophil count on admission (mean ± SD)	206.9 ± 139.8
Minimal eGFR (mean ± SD)	62.7 ± 41.5
Maximal C-reactive protein (median, IQR)	10.3 ± 6.9
Platelet count on admission (mean ± SD)	382.0 ± 152.9
Maximal platelet count during follow-up (mean ± SD)	400.4 ± 126.9
Outcomes	
Rash N (%)	0 (0)
Treatment duration in days, median, IQR	42, 40-52
Length of hospitalization stay, median, IQR	27, 16-35
Recurrent hospitalization within 6 months of discharge N (%)	7 (53.8)
